# Oxypaeoniflorin Prevents Acute Lung Injury Induced by Lipopolysaccharide through the PTEN/AKT Pathway in a Sirt1-Dependent Manner

**DOI:** 10.1155/2021/6878026

**Published:** 2021-08-04

**Authors:** Fan Guohua, Zhu Tieyuan, Wang Rui, Xiong Juan

**Affiliations:** ^1^Department of Thoracic Surgery, Renmin Hospital of Wuhan University, Jiefang Road 238, Wuhan 430060, China; ^2^Department of Emergency, Renmin Hospital of Wuhan University, Jiefang Road 238, Wuhan 430060, China

## Abstract

Acute lung injury (ALI) is featured by pulmonary edema, alveolar barrier injury, inflammatory response, and oxidative stress. The activation of Sirt1 could relieve lipopolysaccharide- (LPS-) induced murine ALI by maintaining pulmonary epithelial barrier function. Oxypaeoniflorin (Oxy) serves as a major component of *Paeonia lactiflora* Pall., exerting cardioprotection by activating Sirt1. However, the role of Oxy in ALI induced by LPS remains unclear. The aim of the present study is to illustrate the modulatory effects and molecular mechanisms by which Oxy operates in ALI induced by LPS. The intraperitoneal injection of LPS was performed to establish the murine ALI model while LPS-treated alveolar epithelial cells were used to mimic the *in vitro* ALI model. Levels of lung injury, oxidative stress, and inflammatory response were detected to observe the potential effects of Oxy on ALI. Oxy treatment mitigated lung edema, inflammatory response, and oxidative stress in mouse response to LPS, apart from improving 7-day survival. Meanwhile, Oxy also increased the expression and activity of Sirt1. Intriguingly, Sirt1 deficiency or inhibition counteracted the protective effects of Oxy treatment in LPS-treated mice or LPS-treated alveolar epithelial cells by regulating the PTEN/AKT signaling pathway. These results demonstrated that Oxy could combat ALI *in vivo* and *in vitro* through inhibiting inflammatory response and oxidative stress in a Sirt1-dependent manner. Oxy owns the potential to be a promising candidate against ALI.

## 1. Introduction

Acute lung injury (ALI) and acute respiratory distress syndrome (ARDS) serve as the major cause of death in patients admitted to intensive care unit, giving rise to serve socioeconomic burden [1]. The pathologic changes of ALI are featured by severe hypoxemia, rapid pneumonedema and alveolar injury, uncontrolled inflammatory response, and inflammatory factor accumulation within alveolar space and interstitial tissue [2, 3]. At present, there are still no ideal treatments, although great achievements have been made on the mechanisms underlying ALI [4]. Hence, exploring and developing effective curative candidates are of great importance to prevent and combat ALI.

Silencing information regulator 2-related enzyme 1 (Sirt1) is a deacetylase at the consumption of NAD^+^, which is associated with cell cycle, gene silencing, glucose and fat metabolism, oxidative stress, and aging [5]. Recent studies have unveiled that Sirt1 activation exerted protection in ALI by blocking oxidative stress, inflammation, and apoptosis of pulmonary epithelial cells while Sirt1 knockout can aggravate ALI by activating inflammatory response [6–8]. Based on these findings, exogenous drugs which could activate Sirt1 are promised to combat ALI in the future.

*Paeonia lactiflora* Pall. is a traditional Chinese herbal medicine belonging to the Ranunculaceae family, which possesses the ability to reduce pain, relieve abdominal cramps, and improve blood circulation [9, 10]. Oxypaeoniflorin (Oxy) and paeoniflorin are two major active components in *Paeonia lactiflora*, both of which could exert anti-inflammatory and antiapoptotic effects [11, 12]. In addition, as a monoterpene glycoside compound, Oxy could also inhibit advanced glycation end product-induced oxidative damage in mesangial cells [13]. One recent study also reported that Oxy improved myocardial ischemia/reperfusion (I/R) injury via activating the Sirt1/forkhead transcription factor FKHR (Foxo1) signaling pathway [12]. However, previous studies paid little attention to the beneficial effects in Oxy on ALI. Therefore, in the present study, we intended to investigate whether Oxy treatment could prevent ALI and meanwhile to unveil the possible mechanisms of Oxy in ALI.

## 2. Materials and Methods

### 2.1. Reagents

Oxypaeoniflorin (Oxy) (purity > 98.06%) was purchased from MedChemExpress (#HY-N0748, Shanghai, China). Fetal bovine serum (FBS), trypsin-EDTA (0.25%) phenol red, and Dulbecco's modified Eagle's medium, Nutrient Mixture F-12 (DMEM/F-12), were purchased from Invitrogen-Gibco (Grand Island, NY). Anti-GAPDH (ab8245), anti-Sirt1 (ab189494), anti-Bcl-2 (ab32124), anti-Bax (ab32503), and anti-4-HNE (ab48506) antibodies were obtained from Abcam (Cambridge, MA, USA). The secondary antibody was obtained from LI-COR Biosciences (Lincoln, United States). The QuantiPro ™ Bicinchoninic Acid Protein Assay Kit from Sigma-Aldrich LLC (St. Louis, MO, USA) was employed to analyze protein concentrations. Lipopolysaccharide (LPS) was obtained from Sigma-Aldrich (#L6386-25MG, Beijing Merck Limited, China). All chemical reagents used were of analytical grade in the present study.

### 2.2. Animals and Animal Model

Male C57BL/6 mice, weighing 23–25 g, were purchased from the Institute of Laboratory Animal Science, Chinese Academy of Medical Sciences. Sirt1 global knockout mice with C57BL/6 background, weighing 23–25 g, were provided by the Model Animal Research Center of Nanjing University. As described [12], Oxy diluted in sterile saline containing DMSO was administered intragastrically at the concentration of 40 mg/kg/day for 30 consecutive days before LPS installation. The sepsis-induced ALI model was constructed by installing LPS intratracheally (5 mg/kg) for 12 hours as reported [14]. The control groups were given an isovolumetric sterile saline. 12 hours after LPS installation, the animals were sacrificed under deep anesthesia by cervical dislocation. Next, the left lungs from 5 mice in each group were excised and instilled with formalin to expand the alveoli. And the left lungs from the other 5 mice in each group were dried in an 80°C oven for 3 days. The lung wet/dry (W/D) ratio was calculated to assess edema. The right lung tissues were stored at -80°C for biochemical analysis.

### 2.3. Alveolar Fluid Clearance

Alveolar fluid clearance in this study was carried out by measuring a progressive increase in the concentration of alveolar Evans blue dye as described [15]. To be more specific, after thoracotomy, the trachea and lung were excised integrally. Subsequently, 5% Evans blue-labeled albumin dissolved in saline solution (1 mL) was perfused into the alveolar space. Then, air (2 mL) was pumped to make the saline fully occupy the alveolar spaces. Next, the lung tissues were stored and inflated with pure oxygen at 37°C for 1 hour, the airway pressure of which was kept at 7 cm H_2_O.

### 2.4. Cell Counts in Bronchoalveolar Lavage Fluid (BALF)

The bronchoalveolar lavage fluid (BALF) was collected as soon as the mice were sacrificed. The lungs were lavaged by ice-cold phosphate buffer saline (1.0 mL) (pH = 7.4) three times to acquire BALF samples. Then, the BALF samples were centrifuged (420 × g) at 4°C for 15 min. Sterile saline (1.0 mL) was used to resuspend cell pellets. Next, 100 *μ*L of each BALF sample was cytocentrifuged in a cytospin for 6 min at 450 rpm. Total cells, macrophages, and neutrophils in BAFL were counted via a hemocytometer and Wright-Giemsa staining double-blindly. Cells were observed (300 cells/slide) under a light microscope.

### 2.5. Cell Culture and Treatment

The MLE-12 lung epithelial cell line was obtained from Kunming Cell Bank of Typical Cultures Preservation Committee, Chinese Academy of Sciences (Kunming, China). The MLE-12 lung epithelial cells were incubated with DMEM supplemented with 10% fetal bovine serum (FBS) in a 5% CO_2_ incubator at 37°C. Sirt small interfering RNA (siRNA) was used to knock down the expression of Sirt1 in lung epithelial cells. The siRNA targeting Sirt was provided by GenePharma Co. Ltd. (Shanghai, China). The cells were transfected using Lipofectamine 2000 (Thermo Fisher Scientific, Waltham, MA, USA) based on the manufacturer's instructions. To mimic LPS-induced lung injury, lung epithelial cells were treated with LPS at the concentration of 100 *μ*g/mL with or without Oxy (10 *μ*M) for 6 h as described [12].

### 2.6. Cell Viability

Cell viability was accessed using the commercial CCK-8 assay based on the manufacturer's instructions.

### 2.7. Quantitative Real-Time RT-PCR

Total RNA of lung tissues or cells was extracted according to the manufacturer's protocol for real-time PCR. The relative RNA quality was determined by UV analysis. After the PCR reaction solution was configured, the PCR was amplified by the real-time PCR machine. The PCR reaction conditions were as follows: predenaturation at 93°C for 2 minutes, then a total of 35 cycles of denaturation at 93°C for 1 minute, annealing at 55°C for 1 minute, and extension at 72°C for 1 minute, finally followed by 72°C for 7 minutes. The PCR product was standardized to the internal reference GAPDH using the 2^−*ΔΔ*Ct^ method. The primers used in our study are presented in Table 1.

### 2.8. Western Blot

Total proteins were extracted for protein detection from mouse lung tissues or cells according to the manufacturer's protocol. Briefly, 200 mg lung tissues were lysed into homogenate by 200 *μ*L RIPA buffer containing phosphatase inhibitor and protease inhibitor. After being ground evenly and incubated on ice for 30 minutes, the homogenate was centrifuged at 12000 rpm for 20 minutes at 4°C. Then, the concentration of the supernatant was determined with a BCA kit. Protein extracts that have added to the loading buffer solution were boiled at 95°C for 10 minutes. Proteins with equal amounts were separated on polyacrylamide gels by electrophoresis and then transferred onto polyvinylidene fluoride (PVDF) membranes. Afterward, membranes were blocked with 5% bovine serum albumin (BSA) at 37°C for 1 hour. And then, the membranes were incubated with primary antibodies at 4°C overnight. After washing 3 times with PBS, the membranes were incubated with the secondary antibody at 37°C for 1 hour. Finally, the protein bands were detected with 2,4-diaminobutyric acid (DAB) chromogenic solution by an enhanced chemiluminescence kit.

### 2.9. Hematoxylin-Eosin (HE) Staining

The lung tissues were excised and fixed in 4% paraformaldehyde solution. The samples were sliced into 5 *μ*m thick sections after embedded in paraffin. Then, sections were dehydrated using xylene, gradient alcohol, and distilled water in sequence. Afterward, the sections were stained with hematoxylin and eosin (HE). Next, the sections were dehydrated using gradient alcohol and xylene and sealed with neutral gum. Finally, histopathology was observed using a microscope. The inflammation score for lung injury was assessed using a semiquantitative scoring system according to the following categories [2]: pulmonary edema, neutrophil infiltration, hemorrhage, and disorganization of lung parenchyma. In briefly, the grading scale as follows: 4+ = very severe injury (almost 100%); 3+ = severe injury (75%); 2+ = moderate injury (50%); 1+ = light injury (25%); 0 = none, was employed to score the degree of lung injury in all samples. The inflammation status was scored by 2 independent pathologists who were blinded to this experiment.

### 2.10. Immunohistochemical Staining

The lung tissue sections washed with PBS were incubated with 3% H_2_O_2_ for 10 minutes in PBS to block endogenous peroxides. Afterward, the sections were added to 10% goat serum blocking solution at room temperature for 20 minutes and incubated with primary antibody against 4-HNE at 4°C overnight. The sections were incubated with secondary antibody at 37°C for 15 minutes after washing. After that, the sections were incubated with Streptavidin Biotin HRP Complex, colored with preprepared DAB chromogenic solutions at room temperature, followed by being stained with hematoxylin for 2 minutes. Finally, the sections were observed using a microscope. Quantification of the immunopositive area was analyzed by ImageJ software as the ratio of the positive-stained area to the total area.

### 2.11. Tunel Staining

The slides of snap-frozen lung tissues were stained using Tunel assay according to the manufacturer's protocol. The samples were sliced into 5 *μ*m thick sections and dewaxed after embedded in paraffin. The sections were treated with proteinase K (10~ 20 *μ*g/mL) for 15 minutes at room temperature and washed with PBS for about 10 seconds. Then, the sections were added to the preprepared reaction solution and react at 37°C in a humidified cabinet for 60~90 minutes. Afterward, the sections were washed with PBS 3 times for 5 minutes each time and observed cells using a microscope. For each group, 2 independent pathologists who were blinded to this experiment evaluated the number of Tunel-positive cells and total number of cells in each microscopic field, aiming to calculate the level of apoptosis in lung tissues.

### 2.12. Statistical Analysis

The data in our study are presented as mean ± SEM and analyzed by SPSS 23.0. Two-group comparisons were carried out by Student's unpaired *t*-test. Two-group comparisons were performed by Student's unpaired *t*-test. *P* < 0.05 was regarded as significantly different.

## 3. Results

### 3.1. Oxy Alleviated LPS-Induced Lung Pathological Injury and Improved Survival of Mice

**Figure 1:**
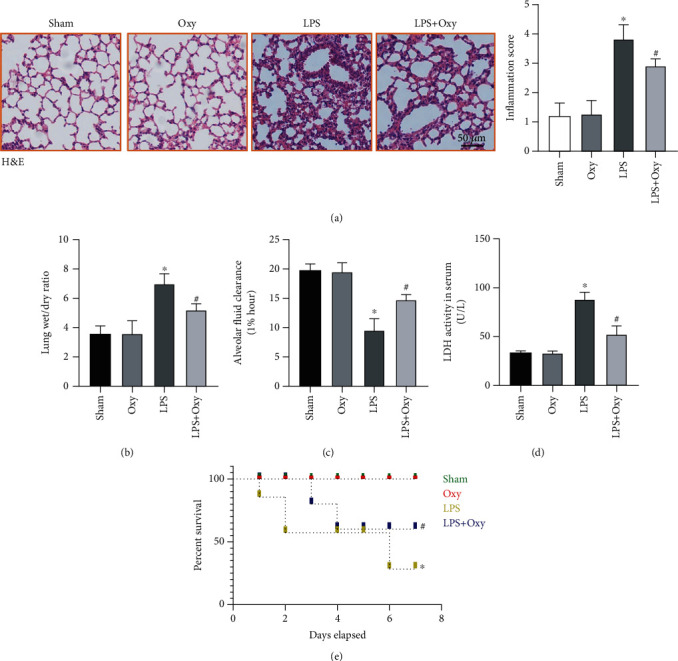
Oxy alleviated LPS-induced lung pathological injury and improved survival of mice. Mice were administrated with Oxy 40 mg/kg/day by intragastrical gavage for 30 days before LPS installation. At 12 h after LPS stimulation, mice were killed and lung tissues were collected. (a) H&E staining for lung tissues. (b) Lung wet/dry ratio. (c) Alveolar fluid clearance. (d) LDH activity in serum. (e) 7-day survival of LPS-treated mice with or without Oxy pretreatment (*n* = 5, ^∗^*P* < 0.05 vs. sham group, ^#^*P* < 0.05 vs. LPS group).

To begin with, we investigated whether Oxy could affect LPS-induced ALI in mice. As shown in Figures 1(a)–1(d), LPS significantly triggered lung pathological injury, as evidenced by higher inflammation score and lung wet/dry compared with the sham group. Meanwhile, LPS stimulation also decreased alveolar fluid clearance and increased the activity of LDH in serum. Oxy pretreatment significantly inhibited pneumonedema and injury of mice with ALI. Also, we can see that Oxy treatment did not trigger lung pathological injury under basal conditions. Finally, we also observed the effect of Oxy on the 7-day survival rate of mice with ALI. As shown in Figure 1(e), Oxy significantly improved the percent survival of mice with ALI compared with the sham group. Collectively, these data showed that Oxy pretreatment not only exerted protective roles in LPS-induced ALI but also improved survival.

### 3.2. Oxy Inhibited LPS-Induced Inflammatory Response in Murine Lung Tissues

**Figure 2:**
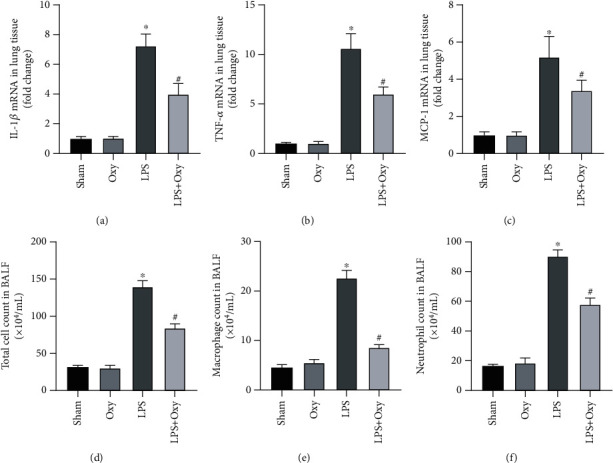
Oxy inhibited LPS-induced inflammatory response in murine lung tissues. (a–c) The mRNA levels of proinflammatory factors including IL-1*β*, TNF-*α*, and MCP-1. (d–f) Total cell, macrophage, and neutrophil count in BALF (*n* = 5, ^∗^*P* < 0.05 vs. sham group, ^#^*P* < 0.05 vs. LPS group).

Inflammatory response is one of the critical features during sepsis-induced ALI [16]. Next, we detected the mRNA levels of proinflammatory genes and the cell counts in BALF. As shown in Figures 2(a)–2(c), Oxy pretreatment could significantly inhibit the upregulation of IL-1*β*, TNF-*α*, and MCP-1 triggered by LPS. In addition, Oxy pretreatment also decreased the total cell count, macrophage count, and neutrophil count in BALF of mice challenged with LPS (Figures 2(d)–2(f)). These data demonstrated that Oxy (40 mg/kg/day for 30 days) displayed significant anti-inflammatory action in lung tissues in response to LPS stimulation. And this concentration of Oxy showed no obvious effects on the levels of IL-1*β*, TNF-*α*, and MCP-1 and cell counts in BALF under basal conditions. These results hinted that Oxy could decrease LPS-induced inflammatory response in mice with ALI.

### 3.3. Oxy Relieved LPS-Induced Oxidative Stress in Murine Lung Tissues

**Figure 3:**
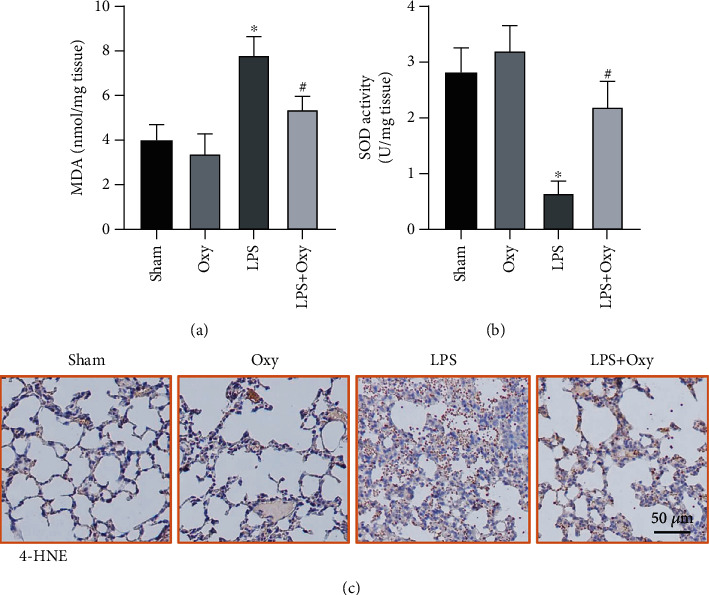
Oxy relieved LPS-induced oxidative stress in murine lung tissues. (a) MDA content in lung tissues. (b) SOD activity in lung tissues. (c) Immunohistochemical staining for 4-HNE (*n* = 5, ^∗^*P* < 0.05 vs. sham group, ^#^*P* < 0.05 vs. LPS group).

Excessive reactive oxygen species induced by LPS could destroy the organic antioxidative defense system and aggravate sepsis-induced ALI by triggering damage to lipids, DNA, and proteins [2]. Hence, we next investigated the roles of Oxy in LPS-induced oxidative stress. As shown in Figures 3(a) and 3(b), the level of advanced lipoxidation end product malonaldehyde (MDA) significantly increased while the level of superoxide dismutase (SOD) decreased in lung tissues after LPS stimulation. And Oxy pretreatment decreased the level of MDA and increased the level of SOD. Immunohistochemical staining further showed that Oxy could inhibit the level of oxidative stress triggered by LPS (Figure 3(c)).

### 3.4. Oxy Decreased LPS-Induced Apoptosis in Murine Lung Tissues

**Figure 4:**
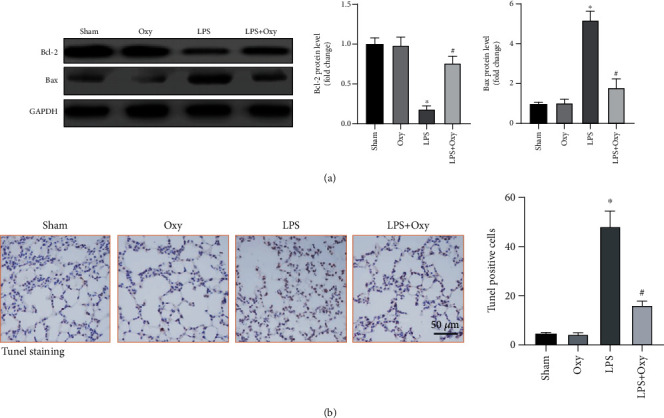
Oxy decreased LPS-induced apoptosis in murine lung tissues. (a) Western blot image and analysis of the protein of Bax and Bcl-2. (b) Tunel staining for apoptotic cells (*n* = 5, ^∗^*P* < 0.05 vs. sham group, ^#^*P* < 0.05 vs. LPS group).

LPS challenge also gives rise to apoptosis of pulmonary epithelial cells, the inhibition of which is important for maintaining pulmonary function [17]. Herein, we detected the makers of apoptosis in LPS-treated lung tissues. Western blots showed that Oxy significantly increased the level of Bcl-2 and decreased the level of Bax in lungs challenged with LPS (Figure 4(a)). Consistently, Tunel staining further proved the antiapoptosis effect of Oxy in ALI (Figure 4(b)). Taken together, the above data robustly verified the vital involvement of the Oxy-mediated antiapoptosis effect.

### 3.5. Sirt1 Was Involved in Oxy-Mediated Protection in ALI

**Figure 5:**
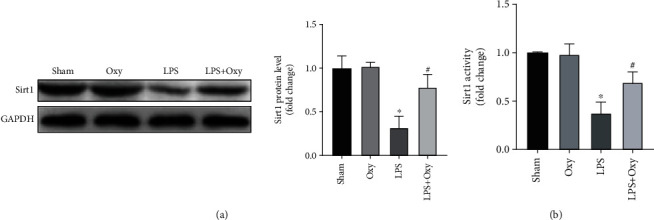
Sirt1 was involved in Oxy-mediated protection in ALI. (a) Western blot image and analysis of the protein of Sirt1. (b) Sirt1 activity (*n* = 5, ^∗^*P* < 0.05 vs. sham group, ^#^*P* < 0.05 vs. LPS group).

A previous study has clarified the critical position of Sirt1 in LPS-induced ALI [18]. Meantime, Oxy could also enhance the expression and activity of Sirt1 in cardiac tissues. We wonder if the protective effects of Oxy are associated with Sirt1 activation. Firstly, we detected the protein expression of Sirt1 in the four groups. The results showed that LPS stimulation significantly decreased the protein level of Sirt1 in lung tissue, which could be upregulated after Oxy pretreatment (Figure 5(a)). As expected, Oxy pretreatment also increased the activity of Sirt1 in lung tissue of mice with ALI (Figure 5(b)). In brief, we speculate that Oxy-mediated protection in ALI is associated with Sirt1 activation.

### 3.6. Oxy Protected against LPS-Induced Inflammation, Oxidative Stress, and Apoptosis of Lung Epithelial Cells in a Sirt1-Dependent Manner *In Vitro*

**Figure 6:**
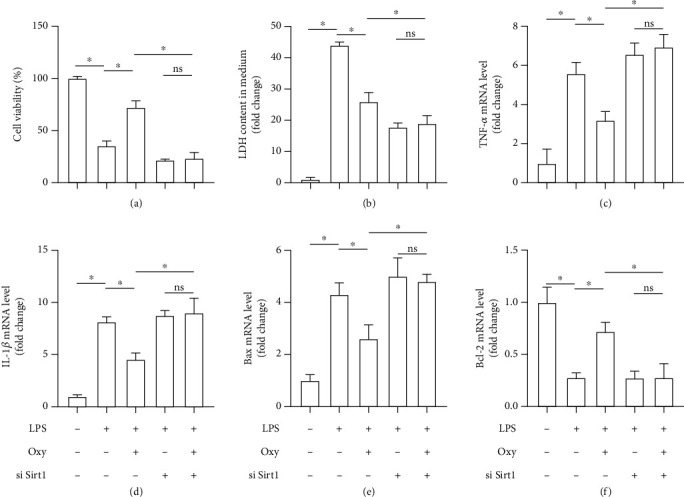
Oxy protected against LPS-induced inflammation, oxidative stress, and apoptosis of lung epithelial cells in a Sirt1-dependent manner *in vitro.* (a) Cell viability detected by the CCK-8 assay kit. (b) LDH content in a medium in the indicated groups. (b) LDH content in a medium in the indicated groups. (c, d) The mRNA levels of proinflammatory factors including TNF-*α* and IL-1*β*. (e, f) The mRNA levels of Bax and Bcl-2 (*n* = 5, ^∗^*P* < 0.05 vs. the indicated groups).

To further verify our hypothesis, we next investigated the roles of Oxy in LPS-treated lung epithelial cells *in vitro* with or without Sirt1 knockdown. As expected, LPS stimulation (6 h) significantly decreased cell viability and increased the levels of LDH, TNF-*α*, and IL-1*β*, apart from promoting apoptosis, all of which could be reversed by Oxy treatment. However, Sirt1 knockout abolished the protective roles of Oxy. Additionally, in the context of Sirt1 knockdown, Oxy also lost its protective effects on LPS-treated lung epithelial cells (Figures 6(a)–6(f)), suggesting that Oxy exerted pulmonary protection in a Sirt1-dependent manner.

### 3.7. Sirt1 Deficiency Abolished the Protective Roles of Oxy in Mice with ALI

**Figure 7:**
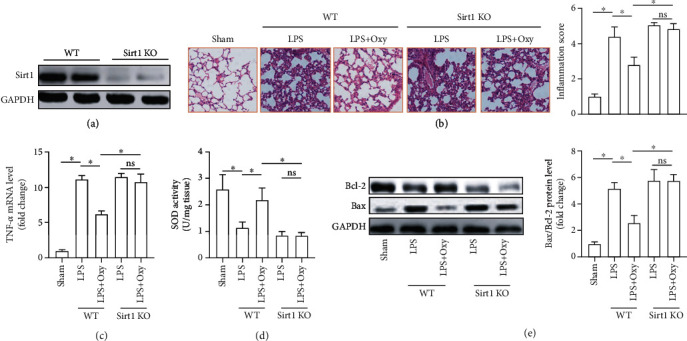
Sirt1 deficiency abolished the protective roles of Oxy in mice with ALI. (a) Western blot image of the protein of Sirt1. (b) H&E staining for lung tissues. (c) The mRNA levels of TNF-*α*. (d) SOD activity in lung tissues. (e) Western blot image and analysis of the protein of Bax and Bcl-2 (*n* = 5, ^∗^*P* < 0.05 vs. the indicated groups).

At last, we used Sirt1 global knockout mice to prove our hypothesis. Sirt1 knockout was confirmed by western blots (Figure 7(a)). H&E staining showed that Oxy could not alleviate pathological injury in Sirt1 deficiency mice challenged with LPS. Meanwhile, Sirt1 deficiency also abolished the protective role of Oxy against LPS-induced pathological injury (Figure 7(b)). Additionally, we found that Sirt1 deficiency also counteracted the antioxidant, anti-inflammatory, and antiapoptotic effects from Oxy in mice with ALI, which was evidenced by the increased TNF-*α*, the decreased SOD activity, and the increased ratio of Bax/Bcl-2 (Figures 7(c)–7(e)). These data further confirmed that the protective roles of Oxy were determined by Sirt1 activation.

### 3.8. Oxy Regulated the PTEN/AKT Pathway in a Sirt1-Dependent Manner during ALI

**Figure 8:**
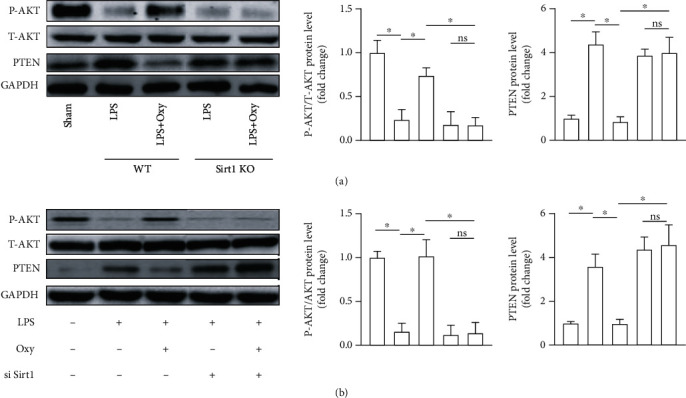
Oxy regulated the PTEN/AKT pathway in a Sirt1-dependent manner during ALI. (a) Western blot image and analysis of the protein of PTEN and AKT in mice with ALI. (b) Western blot image and analysis of the protein of PTEN and AKT in LPS-treated lung epithelial cells (*n* = 5, ^∗^*P* < 0.05 vs. the indicated groups).

The AKT signaling pathway, which could be inhibited by PTEN, exerts a vital role in the regulation of inflammation, apoptosis, and oxidative stress [19, 20]. Previous studies have unveiled that Sirt1 could activate the AKT pathway by decreasing the protein level of PTEN, displaying pulmonary protection [21]. Herein, we detected the PTEN/AKT pathway via western blot. As shown in Figures 8(a) and 8(b), LPS stimulation significantly increased the phosphorylation of AKT and decreased the level of PTEN in lung tissues and lung epithelial cells, which could be reversed after Oxy treatment. Meantime, Sirt1 deficiency or knockdown could counteract the modulating effects of Oxy on the PTEN/AKT pathway, indicating that the pulmonary protection of Oxy may be mediated by the PTEN/AKT pathway in a Sirt1-dependent manner.

## 4. Discussion

*Paeonia lactiflora* Pall. is one of the most common traditional Chinese herbal medicines, which was mainly used for the treatment of liver disease over thousands of years [22]. As the main component isolated from *Paeonia lactiflora* Pall., Oxy displays multiple pharmacological actions including antioxidative stress, antiapoptosis, and anti-inflammation [23] [9, 24]. The significant effects of Oxy on oxidative stress, apoptosis, and inflammation suggest a possible application for Oxy in infection and ALI. However, the relevance of Oxy in LPS-induced ALI was largely unknown. Our study demonstrated that Oxy dramatically alleviated pathological lung injury, epithelial inflammation, oxidative stress, and apoptosis induced by LPS stimuli.

LPS-induced ALI is generally featured by an overwhelming inflammatory response, which triggers the excessive production of inflammatory factors, involving TNF-*α*, IL-1*β*, and monocyte chemoattractant protein-1 (MCP-1) [25]. Subsequently, overactivation of the inflammatory response gives rise to pathological injury of the alveolar epithelium, eventually leading to ALI. Clinical studies have uncovered that the persistent increase of TNF-*α*, IL-1*β*, and MCP-1 in plasma is highly associated with high mortality in patients with ALI [26]. Oxidative stress is also implicated with the development of ALI. When the body suffers from risk factors of ALI, excessive reactive oxygen species involve nonfree radical species such as singlet oxygen (^1^O_2_) and hydrogen peroxide (H_2_O_2_) and free radicals such as hydroxyl radicals (OH^·^) and superoxide anion radicals (O_2_^·−^) [27]. Under physiological conditions, cells express many antioxidant proteins such as SOD, nicotinamide adenine dinucleotide phosphate, glutamate-cysteine ligase catalytic subunit, catalase, quinone-1, glutathione peroxidase, and heme oxygenase-1, which could scavenge intracellular reactive oxygen species [28]. A good deal of reactive oxygen species could give rise to the unsaturation of fatty acids in membranes, alter membrane permeability, and impair membrane fluidity, eventually resulting in lung edema and injury by damaging alveolar epithelial barrier. In addition, lung epithelial apoptosis also participates in the development of ALI induced by LPS. In patients with ALI, the apoptosis of alveolar epithelial cells is enhanced, which impairs microvascular integrity and promotes the release of proinflammatory cytokines [29]. In the present study, we found that Oxy could significantly inhibit alveolar epithelial oxidative stress, apoptosis, and inflammatory response induced by LPS *in vivo* and *in vitro*, eventually alleviating lung injury and improving survival.

Sirt1 is a nicotinamide adenine dinucleotide-dependent histone deacetylase, exerting anti-inflammatory, antiapoptosis, and antioxidative stress effects [15, 30, 31]. Recent studies reported that Sirt1 activation protected against inflammatory pathogenesis of ALI. Hence, some candidates including u-3 PUFA and resveratrol could relieve pulmonary edema and lung injury by activating Sirt1 [32, 33]. Here, we found that Oxy could upregulate and activate Sirt1 in the context of ALI. Notably, under basal conditions, Oxy exhibited no effects on the expression and activity of Sirt1. Meanwhile, we also disclosed that the inhibition of Sirt1 could abolish the protective effects of Oxy on ALI, suggesting that Oxy prevented LPS-induced ALI in a Sirt1-dependent manner. The PI3K/AKT signaling pathway is a critical regulator of cell survival, and PTEN is a multifunctional phosphatase which could negatively regulate the AKT pathway and displays tumor-suppressive effect [20]. Previous studies have shown that activation/phosphorylation of the AKT pathway by Sirt1 could protect against ALI by inhibiting oxidative stress, inflammation, and apoptosis [21, 34]. In the present study, we found that Oxy could inhibit the AKT pathway by activating PTEN in a Sirt1-dependent manner during ALI.

**Figure 9:**
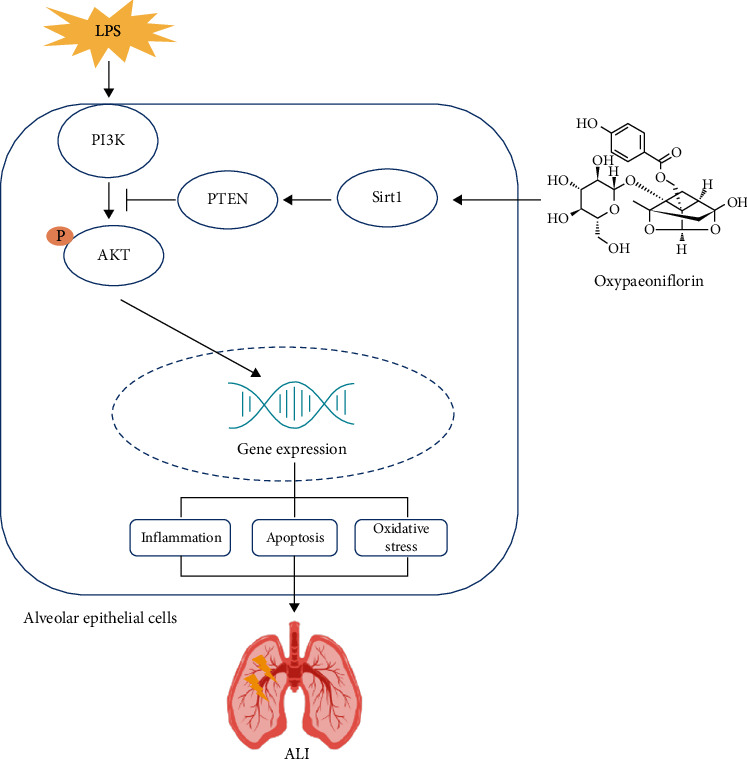
Schematic model describing the possible effect of oxypaeoniflorin on inhibiting oxidative stress, inflammation, and apoptosis of LPS-treated alveolar epithelial cells.

In conclusion, we found that Oxy alleviated LPS-induced ALI via regulating lung epithelial inflammation, apoptosis, and oxidative stress by regulating the PTEN/AKT pathway in a Sirt1-dependent manner (Figure 9). Our study may provide insights for the future treatment of sepsis and ALI via the application of Oxy.

## Figures and Tables

**Table 1:** The primers used in the study.

Species	Gene	Forward primer	Reverse primer
Mice	*Tnf-α*	ACTGAACTTCGGGGTGATCGGT	TGGTTTGCTACGACGTGGGCTA
Mice	*Mcp-1*	CAGCTAGCTCAAACGTAGCCC	ACGATGCTAGTGCTAGTGCTGACC
Mice	*Il-1β*	AATGAAGGAACGGAGGAGCC	CTCCAGCCAAGCTTCCTTGT
Mice	*Bax*	ACGTAGTCGCTAGTCAAACTG	CAGTACCAACGTAGTCGTAGTCG
Mice	*Bcl-2*	AGCTAGTTACGTAGCTGATCGT	ACGTAGTCGTAGTCGTAACCC
Mice	*Gapdh*	ACTCCACTCACGGCAAATTC	TCTCCTATGGTGGTGACGACA

## Data Availability

All data supporting the findings in our study are available from the corresponding author upon reasonable request.
